# Diagnosing Metformin Intoxication with High-Resolution Platelet Respirometry: A Case Report

**DOI:** 10.3390/ijms27104631

**Published:** 2026-05-21

**Authors:** Ondřej Sobotka, Pavla Staňková, Joao Fortunato, Eva Trčková, Pavel Skořepa

**Affiliations:** 1 3rd Department of Internal Medicine—Metabolic Care and Gerontology, University Hospital Hradec Kralove, Sokolská 581, 500 05 Hradec Kralove, Czech Republic; sobotkao@lfhk.cuni.cz (O.S.); joao.fortunato@fnhk.cz (J.F.); eva.trckova@centrum.cz (E.T.); 2 3rd Department of Internal Medicine—Metabolic Care and Gerontology, Faculty of Medicine in Hradec Králové, Charles University, Šimkova 870, 500 03 Hradec Kralove, Czech Republic; 3 Department of Military Internal Medicine and Military Hygiene, Military Faculty of Medicine, University of Defence, 500 03 Hradec Kralove, Czech Republic; 4 Department of Physiology, Faculty of Medicine in Hradec Králové, Charles University, Šimkova 870, 500 03 Hradec Kralove, Czech Republic; stankovap@lfhk.cuni.cz

**Keywords:** metformin, mitochondria, high-resolution respirometry, intoxication

## Abstract

Metformin-associated lactic acidosis (MALA) involves mitochondrial Complex I inhibition, traditionally diagnosed via indirect markers. We present platelet high-resolution respirometry (HRR) as a novel “liquid biopsy” to directly quantify metformin-induced systemic bioenergetic lesions. A 65-year-old diabetic male presented with severe lactic acidosis, acute kidney injury, and profound hypoglycemia after intentionally overdosing on metformin (120 g), dapagliflozin (600 mg), and insulin glargine (300 U). While hemodialysis cleared plasma metformin and resolved the acidosis, refractory hypoglycemia required high-dose IV glucose for over six days. Day 2 platelet HRR revealed severe Complex I inhibition despite significantly decreased plasma metformin, indicating a profound “toxicodynamic lag.” Mitochondrial bioenergetics recovered by Day 7, reflecting natural platelet turnover. The protracted hypoglycemia was driven by a synergistic triad: metformin-inhibited gluconeogenesis, insulin glargine’s prolonged depot effect, and dapagliflozin-induced persistent renal glucose wasting. Platelet HRR has the potential to be a clinically applicable tool to reveal the “hidden” cellular phase of metformin toxicity missed by standard biomarkers. Furthermore, clinicians must anticipate severe, protracted hypoglycemia in mixed overdoses involving SGLT2 inhibitors.

## 1. Introduction

Metformin is the first-line oral therapy for type 2 diabetes (T2D), valued for its efficacy, low cost, and favorable safety profile [1,2]. Its off-label therapeutic use extends to polycystic ovary syndrome (PCOS), prediabetes, and antipsychotic-induced weight gain [3,4]. Metformin is not metabolized and is excreted unchanged by the kidneys, leading to a half-life of 2–6 h that is significantly prolonged in renal impairment [1,5,6]. While therapeutic plasma levels are 0.1–4 mg/L, supraphysiologic levels are associated with metformin-associated lactic acidosis (MALA), a rare but life-threatening complication with high mortality [7,8,9,10,11,12]. Fundamentally, MALA represents an acute systemic bioenergetic crisis caused by profound mitochondrial poisoning [7]. MALA is strongly associated with acute kidney injury, which precipitates drug accumulation [13].

Metformin’s primary therapeutic and toxic effects are mediated by the inhibition of mitochondrial respiratory Complex I (NADH:ubiquinone oxidoreductase) [14,15]. This inhibition impairs the electron transport system (ETS), reduces ATP synthesis, and elevates the cellular AMP/ATP ratio. This, in turn, activates AMP-activated protein kinase (AMPK), a central energy sensor that modulates glucose uptake and suppresses hepatic gluconeogenesis [16,17,18]. While inhibition of Complex I is the most accepted mechanism, another target, mitochondrial glycerophosphate dehydrogenase (mGPDH), has been proposed to contribute to metformin’s anti-gluconeogenic effects by altering the NAD^+^/NADH ratio [19,20,21]. However, the relevance of mGPDH inhibition at clinical concentrations remains debated [22,23].

The diagnosis of MALA currently relies on the clinical picture and high lactate levels in the context of renal failure and metformin use [8,12]. However, this diagnosis is indirect. Although there is a direct measurement of metformin plasma concentration available, it serves only as a pharmacokinetic marker and does not quantify the severity of intracellular toxicity. Due to the universal effect of metformin on the electron transport system (ETS) in various experimental models [22], a functional measurement of mitochondrial respiration is necessary but lacking in standard practice. We hypothesized that high-resolution respirometry (HRR) of circulating platelets [24] could serve as a “liquid biopsy” to directly quantify the systemic bioenergetic lesion of metformin intoxication and track its reversal in real time.

Mitochondrial bioenergetics is increasingly relevant in clinical medicine, especially in conditions involving metabolic dysfunction, critical illness, and drug-induced mitochondrial toxicity [11,25,26]. Mitochondria are central regulators of cellular energy metabolism, converting nutrients into ATP required for physiological functions such as muscle contraction, neurotransmission, and cellular repair [27,28]. Due to the systemic nature of MALA, assessment of isolated platelet respiration offers a potential new way to diagnose mitochondrial toxicity.

Complicating the clinical picture in modern diabetes management is the frequent use of combination therapies. Gliflozins have emerged as a powerful class of antidiabetic agents with benefits that extend beyond glycemic control. This class of drug promotes urinary glucose excretion but has also been linked to improved cardiovascular outcomes and, importantly, enhanced mitochondrial efficiency. According to some studies, SGLT2 inhibitors became a first-line therapy of heart failure [29,30,31]. However, the systemic metabolic impact of these agents during a catastrophic bioenergetic crisis, such as a mixed overdose where mitochondria are simultaneously poisoned by metformin, remains entirely unknown. Adding exogenous insulin to this regimen can further support glucose lowering in patients with advanced β-cell dysfunction. Together, this combined therapy approach targets hyperglycemia through distinct yet synergistic mechanisms—offering potential for optimized metabolic control, reduced insulin burden, and protection against diabetes-related organ damage [32,33].

Here, we present a case of severe intoxication involving metformin, dapagliflozin, and insulin. Severe metformin intoxication may be accompanied by persistent mitochondrial dysfunction not captured by standard biochemical markers. We therefore explored platelet mitochondrial respirometry as a functional approach to assessing metformin-related bioenergetic toxicity and its recovery over time in severe mixed intoxication.

## 2. Case Presentation

A 65-year-old male with a history of type 2 diabetes mellitus, chronic kidney disease (CKD G3a), ischemic heart disease, recurrent strokes, and depressive disorder presented to the emergency department with generalized weakness, pre-syncope, and marked fatigue. Following initial stabilization, the patient revealed a deliberate suicide attempt 4 h prior to the admission to the emergency department (ED). He reported ingesting approximately 120 tablets of a fixed-dose combination of metformin and dapagliflozin (1000 mg/5 mg each), totaling 120 g of metformin and 600 mg of dapagliflozin, alongside approximately 300 units of insulin glargine U300.

On admission, the patient was somnolent (GCS 13) with signs of circulatory shock. Initial laboratory tests revealed profound hypoglycemia at 1.5 mmol/L (27 mg/dL), lactic acidosis (pH 7.2; Lactate 11.2 mmol/L), and acute kidney injury (Creatinine 209 µmol/L, Table 1).

**Figure 1:**
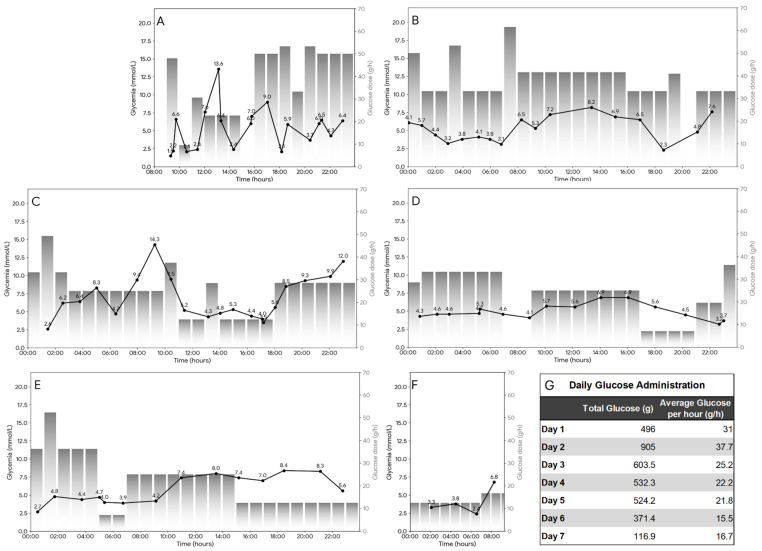
Temporal dynamics of refractory hypoglycemia and parenteral glucose requirements. (**A**–**F**) Daily profiles of plasma glucose concentration (solid black line, left Y axis) and hourly i.v. glucose dose (gray bars, right Y-axis) over the first six days of hospitalization. Panel (**A**) depicts the day of admission (Day 1), followed by the subsequent clinical course in Days 2–6 (**B**–**F**). Note the persistent hypoglycemic excursions requiring high-dose i.v. glucose supplementation, particularly during nocturnal intervals. (**G**) Quantitative summary of parenteral glucose administration. The table lists the total glucose mass administered per day and the calculated average hourly infusion rate, highlighting the massive exogenous carbohydrate requirement needed to counteract the combined toxicity of insulin glargine and dapagliflozin.

**Figure 2:**
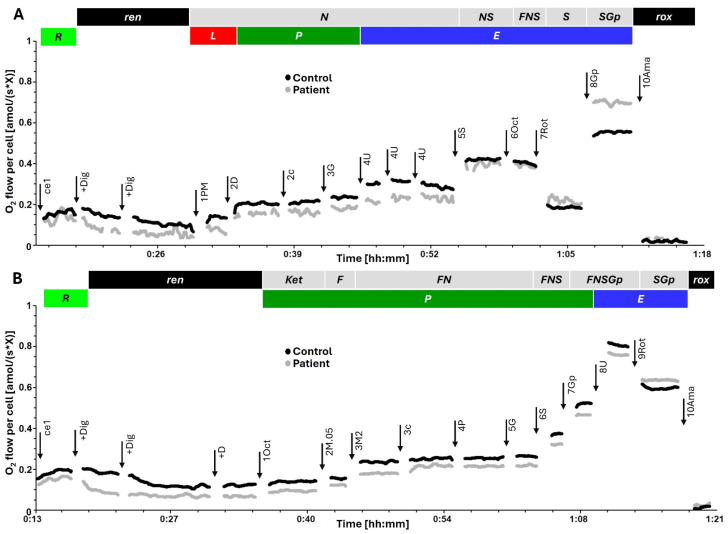
Representative traces of HRR and Substrate–Uncoupler–Inhibitor Titration (SUIT) protocols. Representative traces of O_2_ flow per cell [amol/(s*X)] (non-corrected for instrumental background O_2_ flux) in platelets of control (black) and patient (gray). Titrations are indicated by arrows. Titration spikes were eliminated. (**A**) Sequence of respiratory states in Reference protocol 1 (RP1), characterized by titrations and corresponding rates (routine respiration R of living cells (100 · 10^6^ x/mL); +Dig, digitonin 10 mg mL^−1^; permeabilization of plasma membrane, residual endogenous respiration ren. 1 PM, pyruvate 5 mM and malate 2 mM; N-pathway leak respiration N{PM}L. 2D, ADP 2.5 mM; N-pathway OXPHOS capacity N{PM}P. 2c, cytochrome c 10 µM; N{PM}[c]P; test of mtOM integrity. 3U4, uncoupler titrations to optimum CCCP concentration 4 µM; NADH-linked ET capacity N{PM}E. 4G, glutamate 10 mM; N{PGM}E. 5S, succinate 10 mM; NS-pathway ET capacity NSE. 6Oct, octanoylcarnitine 0.5 mM; FNS-pathway ET capacity FNSE. 7Rot, rotenone 0.5 mM inhibiting CI; succinate-pathway ET capacity SE. 8Gp, glycerophosphate 10 mM; SGpE. 9Ama, antimycin A 2.5 µM inhibiting CIII; residual oxygen consumption rox. (**B**) Sequence of respiratory states in Reference protocol 2 (RP2): ce1, R. +Dig, ren. +D, stimulating ren. 1Oct; stimulating ketogenesis. 2M.05, malate 0.05 mM, F-pathway OXPHOS capacity FP = J(1Oct(c)) − J(+M.05); 3M2, malate 2 mM supporting the anaplerotic N-pathway F(N)P; 3c, test of mtOM integrity; 4P, FN{PM}P. 5G, FN{PGM}P. 6S, FNSP. 7Gp, FNSGpP. 8U4, uncoupler titrations to optimum CCCP concentration 4 µM; FNSGpE. 9Rot, SGpE. 10Ama, rox.

After the initial infusions of glucose in the ED, the patient still had tendencies for hypoglycemia (Figure 1) and drop in blood pressure. Moreover, a toxic plasma metformin concentration of 31 mg/L (242 µM) was subsequently confirmed, and the patient was transferred to the medical intensive care unit (ICU). Noradrenaline was initiated for circulatory failure, and intermittent hemodialysis (IHD) was performed immediately (initiated approximately 6 h post-admission) to enhance metformin elimination. To assess the cellular extent of mitochondrial toxicity, platelet mitochondrial respiration was measured in the morning of day 2, approximately 12 h after the conclusion of the IHD session. Despite the resolution of systemic lactic acidosis and elimination of metformin from the body, functional analysis revealed persistent, severe inhibition of mitochondrial respiration (Figure S1 and Figure 2). This finding suggested that intracellular toxicity persisted despite plasma clearance. While the lactic acidosis was resolved following the initial dialysis sessions, and noradrenaline support was discontinued 24 h after admission, the hypoglycemia proved refractory (Figure 1). Due to the massive overdose of long-acting insulin glargine and the persistent glycosuric effect of dapagliflozin, the patient required continuous high-dose glucose infusions (20% dextrose) for next six days (Figure 1). Approximately 72 h after ICU admission, insulinemia remained exceptionally high (3385.8 mU/L; reference 2.4–24.0 mU/L) with a suppressed C-peptide (139 pmol/L), confirming the exogenous source and the prolonged half-life of the depot insulin in the setting of renal injury. Following metabolic stabilization, resolution of the mitochondrial defect, and psychiatric consultation, he was transferred to a psychiatric department for further care.

## 3. Results

**Figure 3:**
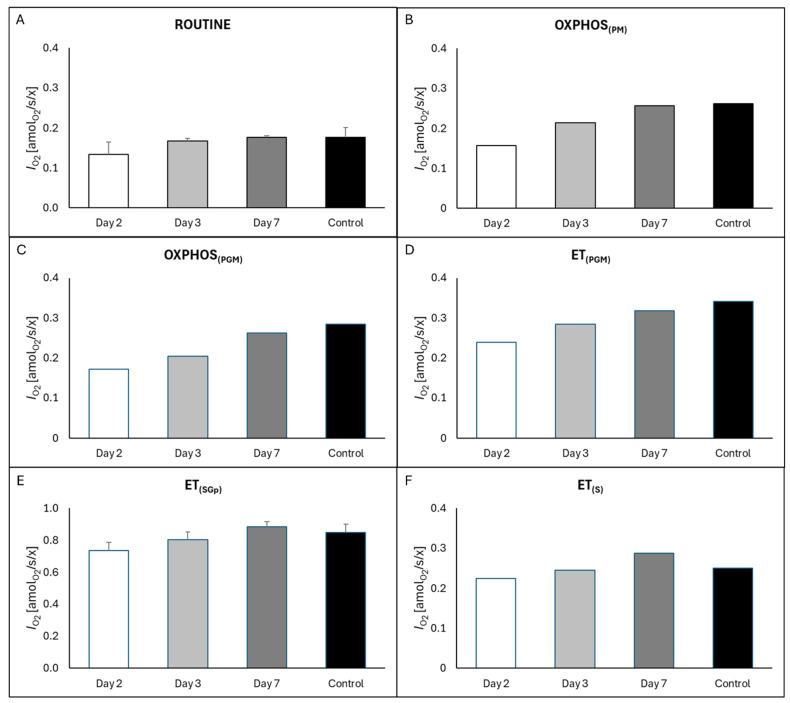
Inhibition and recovery of platelet mitochondrial respiration. Quantitative assessment of mitochondrial function in the patient (Days 2, 3, and 7 post-intoxication) compared to a healthy control (Male, 37 y). Data are presented as oxygen flow per unit. (**A**) ROUTINE respiration: Physiological respiration in intact cells, showing severe suppression on Day 2. (**B**,**C**) N-pathway OXPHOS capacity: Capacity supported by NADH-linked substrates, initiating electron transfer via Complex I. (**D**) Maximal noncoupled respiration (ET state) by NADH-linked substrates. (**E**) SGp-pathway capacity: Combined electron transfer capacity of the succinate and glycerophosphate pathways. (**F**) S-pathway capacity: Succinate-linked respiration in the presence of rotenone. ROUTINE respiration and ET(SGp) are average of RP1 and RP2 ± SD.

Overall results of respiratory profiles of platelets isolated from the patient in different days post intoxication and healthy control are shown in Supplementary Figure S1. On Day 2 (post-IHD), high-resolution respirometry HRR revealed a profound inhibition of mitochondrial respiration despite the clearance of plasma metformin. This inhibition was notable already in physiological ROUTINE respiration (R; Figure 3A) and most profound in N-pathway OXPHOS capacity (NP; Figure 3B,C), indicating a specific blockade of Complex I-linked electron transfer. In contrast, electron transfer capacity (E, Figure 3D) and combined S- and Gp-pathway capacity (SGpE) were less compromised (Figure 3E).

Longitudinal monitoring demonstrated a rapid recovery of mitochondrial bioenergetics. By Day 3, respiratory fluxes had partially normalized, and they improved completely on Day 7 (Figure 3). An interesting finding was observed while utilizing succinate as a substrate. While the S-pathway capacity alone (SE; Figure 3F) and combined S- and Gp-pathway capacity (SGpE; Figure 3E) appeared less inhibited than the N-pathway on Day 2, S-pathway capacity reached values comparable to the control on Day 7. The slight elevation observed requires subsequent investigation in larger cohorts to determine if it represents biological variability or adaptive overexpression. However, the comparative interpretation of this result is limited to the character of this report (one patient case report compared to one control individual). Nevertheless, the qualitative pattern of N-pathway specific inhibition is consistent with the known mechanism of metformin toxicity on Complex I [7,22,23], and further studies are required to define potentially other mitochondrial targets and diagnostic patterns. Table 2 details the hematological platelet parameters during the clinical course. Platelet counts remained within the standard reference range across all measurement days, validating the normalization of O_2_ flux per cell.

## 4. Discussion

This case report of severe combined intoxication with metformin, dapagliflozin, and insulin glargine highlights two clinically relevant findings: first, that mixed antidiabetic overdose may produce prolonged and synergistic metabolic toxicity, and second, that HRR of isolated platelets may provide functional information on mitochondrial injury beyond standard biochemical markers.

The clinical picture was dominated by refractory hypoglycemia, which persisted long after the correction of lactic acidosis. This was driven by a specific pharmacological triad. First, metformin compromised the hepatic gluconeogenesis, disabling the body’s primary counter-regulatory mechanism against hypoglycemia. Second, the massive overdose of insulin glargine created a prolonged depot effect. Third, and crucially, dapagliflozin prevented renal glucose reabsorption. Normally, hypoglycemia suppresses urinary glucose excretion; however, SGLT2 inhibition “locked” the kidneys in a glucose-wasting state [29,33]. While SGLT2 inhibitors are increasingly recognized for their long-term mitochondria-protective properties, in the context of an acute energetic crisis and Complex I blockade, this potential benefit was entirely eclipsed by the severe systemic nutrient deprivation induced by continuous renal glucose wasting. This mechanism explains the massive glucose requirements (>7 days) observed in our patient and highlights that in mixed overdoses involving gliflozins, clinicians must anticipate protracted hypoglycemia that defies standard kinetics.

The relationship between metformin and renal failure is often described as accumulation leading to toxicity. The patient presented with acute kidney injury (AKI). Although dialysis efficiently removed the circulating drug, renal parameters did not immediately normalize during the phase of ongoing mitochondrial inhibition on day 2 and 3 (Table 1). Since renal tubular cells are rich in mitochondria and rely heavily on oxidative phosphorylation for active transport, we hypothesize that metformin-induced mitochondrial inhibition may contribute to the persistence of AKI, but functional renal bioenergetic data are absent in this case. Consequently, this specific organ-level mechanism requires testing in targeted models. Because metformin impairs mitochondrial respiration in multiple tissues, ongoing toxicity may promote severe, life-threatening lactic acidosis. The patient received continuous noradrenaline infusion during the Day 2 measurement. Noradrenaline activates platelets via specific adrenergic receptors, altering baseline cellular O_2_ consumption. Day 2 bioenergetic phenotype may partially reflect vasopressor-induced platelet activation combined with toxicological inhibition. Vasopressor support was discontinued prior to Day 3 measurement, eliminating catecholamines as a confounding variable for the recovery timepoints.

Our data reveals a dissociation between plasma pharmacokinetics and cellular pharmacodynamics. On Day 2, plasma metformin levels had dropped significantly, and lactate was normalizing (<2 mmol/L). Yet, high-resolution respirometry (HRR) demonstrated that platelet mitochondria remained inhibited. This “toxicodynamic lag” (hysteresis) confirms that plasma clearance does not equate to cellular recovery. Metformin accumulates within the mitochondrial matrix up to 1000-fold relative to plasma, driven by the membrane potential [15]. Our findings validate platelet HRR as a viable “liquid biopsy” to visualize this trapped toxicity. Unlike plasma lactate, which is a non-specific downstream metabolite, platelet bioenergetics assessment offers a direct readout of the fundamental pathophysiological lesion.

A novel aspect of this report is the longitudinal tracking of mitochondrial recovery. The restoration of respiratory function by Day 7 raises the question of the underlying mechanism. Two hypotheses are currently in question: (1) intracellular washout of the drug and (2) cell turnover. Given that platelets are anucleate and lack extensive machinery for organelle repair or biogenesis, it is unlikely that the “poisoned” platelets repaired their Complex I function. Furthermore, the strong electrostatic retention of metformin suggests that passive diffusion out of the mitochondria is slow. We hypothesize that respiratory recovery by Day 7 reflects physiological platelet turnover. The laboratory data demonstrate an increase in Mean Platelet Volume (MPV) from Day 3 (10.7 fL) to Day 7 (11.1 fL). Larger platelet volume is consistent with the release of younger platelets, but it remains an indirect surrogate marker. We did not quantify the immature platelet fraction (IPF) or reticulated platelets, therefore we cannot definitively confirm accelerated thrombopoiesis. This mechanism remains speculative and requires validation in prospective toxicological models.

This case report possesses significant limitations. The primary limitation of this report is the use of a single age-mismatched control subject. While we previously published a normative dataset of 29 healthy individuals using an identical isolation method [24], that study employed a different high-resolution respirometry titration protocol. Direct numerical comparison of O_2_ flux values between these protocols is invalid. The control data presented serve strictly an illustrative purpose. Future studies must establish protocol-specific institutional reference intervals. Although the observed predominance of N-pathway inhibition is biologically consistent with the established Complex I-directed mechanism of metformin toxicity, these data are insufficient to define a specific respiratory signature, and broader studies will be needed to determine whether additional pathway-level changes are reproducible. We do not have a definitive explanation for the relatively higher S and Gp-pathway values in this patient. Nevertheless, the main contribution of this case report is not the definition of a unique bioenergetic pattern, but the demonstration that platelet respirometry is feasible, minimally invasive, and clinically applicable as a functional measure of mitochondrial toxicity.

## 5. Materials and Methods

### 5.1. Ethics and Consent

The study was conducted in accordance with the Declaration of Helsinki and was approved by the Ethical committee under reference number ‘202505 P09’. The patient was included in the case report after being fully informed and providing written consent for the publication of anonymized clinical data. Informed consent was also obtained from the healthy control individual.

### 5.2. Platelet Isolation

Blood sampling occurred consistently between 08:00 and 09:00 for all reported days. This standardized collection time controls for potential circadian fluctuations in mitochondrial respiratory capacity. Platelet isolation commenced within two hours of blood collection. Platelet isolation was performed according to the density gradient centrifugation (DC) protocol adapted from [24]. Venous blood (12 mL) was collected into K2EDTA tubes. The blood was diluted 1:1 with sterile Dulbecco’s phosphate-buffered saline (DPBS) and layered onto Ficoll-Paque^TM^ in 50 mL Leucosep^TM^ tubes (Greiner Bio-One GmbH, Kremsmünster, Austria). The tubes were centrifuged at 1000× *g* for 10 min at room temperature (RT) without braking. The platelet-enriched buffy coat was harvested, washed with DPBS, and centrifuged at 120× *g* for 10 min to pellet contaminating peripheral blood mononuclear cells (PBMCs). The platelet-rich supernatant was transferred to a fresh tube containing 10 mmol/L EGTA and centrifuged at 1000× *g* for 10 min to pellet the platelets. The final pellet was washed and gently resuspended in 0.5 mL DPBS containing 10 mmol/L EGTA. High-resolution respirometry analysis began immediately following isolation to ensure maximum cell viability and to prevent artifactual respiratory depression.

### 5.3. High-Resolution Respirometry (HRR)

Mitochondrial respiration was measured at 37 °C using an Oroboros O2k (Oroboros Instruments, Innsbruck, Austria). Platelets (100·10^6^ x/mL) were added to 2.0 mL chambers containing mitochondrial respiration medium (MiR05). Data was recorded in real time using DatLab 7.4 software. Oxygen flow JO2 was expressed per cell (amolO2/s/x). Respiration was corrected for residual oxygen consumption (ROX) following the inhibition of Complex III with antimycin A. Two specific Substrate–Uncoupler–Inhibitor Titration (SUIT) protocols were utilized (Reference protocol 1 (RP1) and modified reference protocol 2 (RP2)) to comprehensively map multiple electron entry points, allowing us to isolate the specific Complex I defect from other potential bioenergetic bypasses. We assessed NADH-linked (N-pathway), succinate-linked (S-pathway), glycerophosphate-linked (Gp-pathway) respiration and fatty acid oxidation (F-pathway). For details see the representative measurements in Figure 2. Mitochondrial respiration of permeabilized platelets was measured on day 2, day 3, and day 7 post-ingestion and compared to a single healthy control individual (*N* = 1) measured in three different time intervals (*n* = 3) in parallel O2k chambers. While age and comorbidities may influence baseline respiration rates, they do not account for acute catastrophic bioenergetic collapse; moreover, the patient serves as his own internal control through longitudinal monitoring over the 7-day period.

## 6. Conclusions

Platelet high-resolution respirometry identified a specific and persistent Complex I-linked mitochondrial blockade despite biochemical improvement after severe metformin intoxication. This observation confirms a toxicodynamic lag between macroscopic plasma clearance and microscopic cellular recovery. Concurrently, the clinical course demonstrates the potential for markedly prolonged hypoglycemia in mixed overdoses involving insulin glargine and SGLT2 inhibitors.

## Figures and Tables

**Table 1:** Biochemical parameters at presentation and during the early clinical course.

Parameter	Admission	12 h	Day 2	Day 3	Day 7
Lactate [mmol/L]	11.6	4.1	1.8	0.9	N/A
pH	7.204	7.423	7.45	7.449	7.435
HCO_3_^−^ [mmol/L]	15.9	23.6	26.4	27	23.4
Glycemia [mmol/L]	1.5	6.4	4.1	8.3	10.8
Creatinine [µmol/L]	209	217	144	178	108
eGFR [mL/s]	0.461	0.44	0.723	0.56	1.024
Metformin [mg/L]	31.33	N/A	3.62	N/A	N/A
ROUTINE [amol/(s.X)]	N/A	N/A	0.133	0.167	0.169
OXPHOS_PM_ [amol/(s.X)]	N/A	N/A	0.156	0.213	0.243

Diagnostic indicators of combined intoxication and metabolic derangement. Plasma concentrations of metabolites and drugs were determined from venous blood samples upon admission to the Emergency Department (ED) and prior to hemodialysis (pre-IHD). IHD, intermittent hemodialysis—marked by double line. N/A, not available. ROUTINE indicates physiological O_2_ consumption supported by endogenous substrates in intact cells. OXPHOS_PM_ indicates oxidative phosphorylation capacity supported by externally titrated saturation concentrations of ADP, pyruvate and malate in permeabilized cells.

**Table 2:** Platelet parameters during the clinical course.

Parameter	Ref. Range	Day 2	Day 3	Day 7
Platelet Count [10^9^ x/L]	150–400	203	173	216
Mean Platelet Volume (MPV) [fL]	7.8–11.0	10.9	10.7	11.1
Platelet Distribution Width (PDW) [fL]	9.0–17.0	12.7	12.2	12.2

## Data Availability

Data will be provided upon email request.

## Supplementary Materials

The following supporting information can be downloaded at https://www.mdpi.com/article/10.3390/ijms27104631/s1.

## Abbreviations

The following abbreviations are used in this manuscript:

AKI	acute kidney injury
AMPK	AMP-activated protein kinase
ATP	adenosine triphosphate
CKD	chronic kidney disease
ETS	electron transport system
GCS	Glasgow Coma Scale
HRR	high-resolution respirometry
IHD	intermittent hemodialysis
MALA	metformin-associated lactic acidosis
mGDPH	mitochondrial glycerophosphate dehydrogenase
MiR05	mitochondrial respiration medium
NADH	nicotinamide adenine dinucleotide
OXPHOS	oxidative phosphorylation
ROX	residual oxygen consumption
SGLT2	sodium-glucose cotransporter 2
SUIT	Substrate–Uncoupler–Inhibitor Titration
T2D	type 2 diabetes
