# Supplementary material for: Diagnosing Metformin Intoxication with High-Resolution Platelet Respirometry: A Case Report
Source: Int J Mol Sci. 2026 May 21;27(10):4631. doi: 10.3390/ijms27104631 (PMC13206949; doi:10.3390/ijms27104631)
Supplement: Supplementary file 1 [file ijms-27-04631-s001.zip › ijms-4297964-supplementary.pptx]

## Slide 1
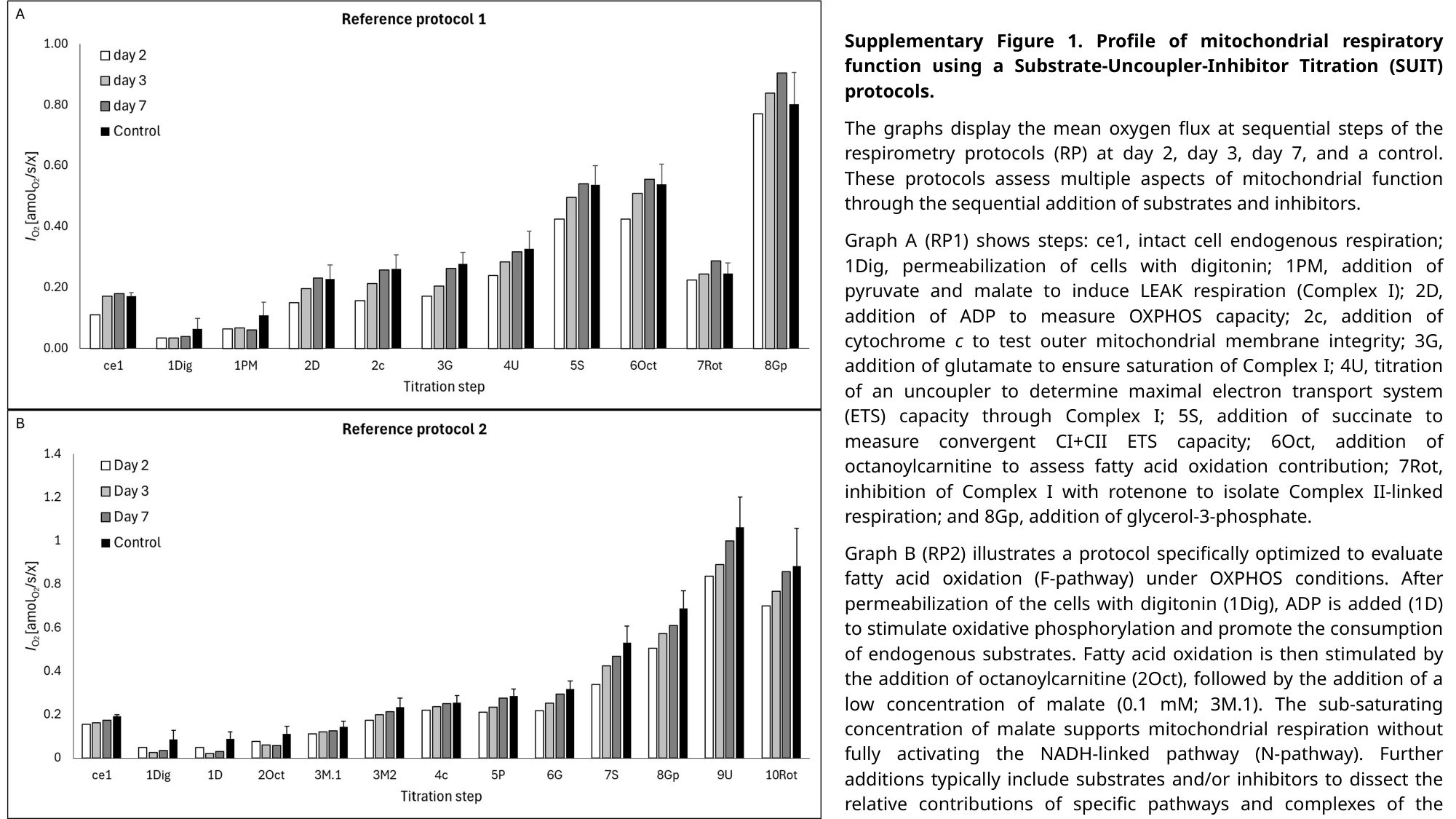

Supplementary Figure 1. Profile of mitochondrial respiratory function using a Substrate-Uncoupler-Inhibitor Titration (SUIT) protocols.
The graphs display the mean oxygen flux at sequential steps of the respirometry protocols (RP) at day 2, day 3, day 7, and a control. These protocols assess multiple aspects of mitochondrial function through the sequential addition of substrates and inhibitors.
Graph A (RP1) shows steps: ce1, intact cell endogenous respiration; 1Dig, permeabilization of cells with digitonin; 1PM, addition of pyruvate and malate to induce LEAK respiration (Complex I); 2D, addition of ADP to measure OXPHOS capacity; 2c, addition of cytochrome c to test outer mitochondrial membrane integrity; 3G, addition of glutamate to ensure saturation of Complex I; 4U, titration of an uncoupler to determine maximal electron transport system (ETS) capacity through Complex I; 5S, addition of succinate to measure convergent CI+CII ETS capacity; 6Oct, addition of octanoylcarnitine to assess fatty acid oxidation contribution; 7Rot, inhibition of Complex I with rotenone to isolate Complex II-linked respiration; and 8Gp, addition of glycerol-3-phosphate.
Graph B (RP2) illustrates a protocol specifically optimized to evaluate fatty acid oxidation (F-pathway) under OXPHOS conditions. After permeabilization of the cells with digitonin (1Dig), ADP is added (1D) to stimulate oxidative phosphorylation and promote the consumption of endogenous substrates. Fatty acid oxidation is then stimulated by the addition of octanoylcarnitine (2Oct), followed by the addition of a low concentration of malate (0.1 mM; 3M.1). The sub-saturating concentration of malate supports mitochondrial respiration without fully activating the NADH-linked pathway (N-pathway). Further additions typically include substrates and/or inhibitors to dissect the relative contributions of specific pathways and complexes of the electron transport chain, analogous to RP1.Data for control are presented as mean ± SD, with oxygen flux normalized per unit of sample (amol/(s·x)).

## Slide 2
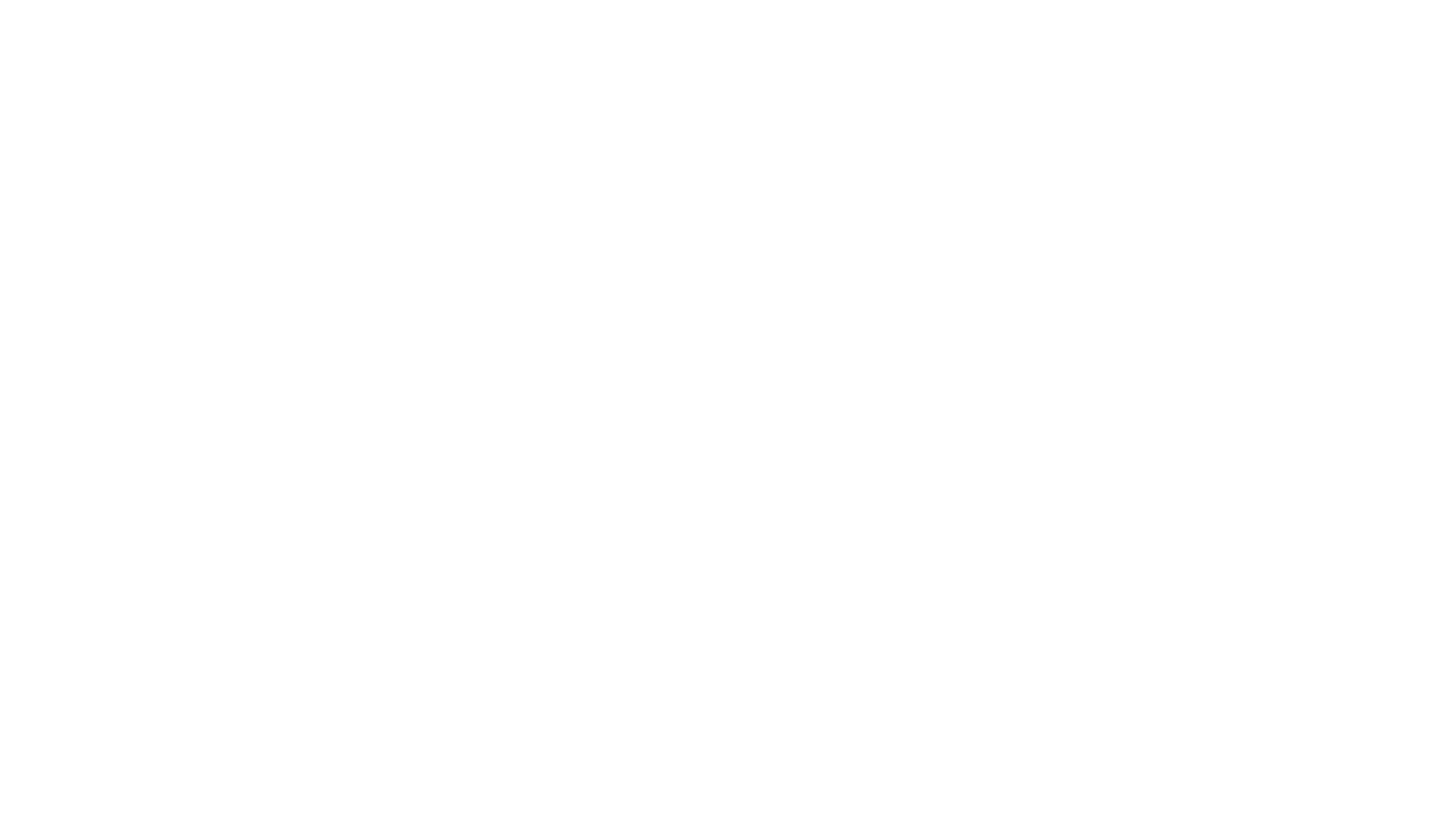

#
